# From Fork to Brain: The Role of AGE–RAGE Signaling and the Western Diet in Neurodegenerative Disease

**DOI:** 10.3390/neurosci6030089

**Published:** 2025-09-09

**Authors:** Haylie J. Pomroy, Arjun Mote, Simeon Mathew, Stebin Chanasseril, Victor Lu, Amanpreet K. Cheema

**Affiliations:** 1 Institute for Neuro Immune Medicine, Dr. Kiran C. Patel College of Osteopathic Medicine, Nova Southeastern University, Fort Lauderdale, FL 33328, USA; 2 Halmos College of Arts and Sciences, Nova Southeastern University, Fort Lauderdale, FL 33328, USA

**Keywords:** advanced glycation end products, receptor for AGEs, neurodegenerative diseases, chronic inflammation, myalgic encephalomyelitis or chronic fatigue syndrome, long COVID, Western diet, Maillard reaction, post-viral neuroinflammatory disease

## Abstract

Advanced glycation end products (AGEs) are reactive compounds formed through non-enzymatic glycation in a process known as the Maillard reaction. While humans produce AGEs endogenously, these compounds can also enter the body through dietary sources, food preparation methods, and exposure to agricultural and food-related chemicals. AGEs can accumulate within cells and impair cellular function. In addition, when AGEs bind to receptors for advanced glycation end products (RAGE), they activate intracellular signaling pathways that promote the generation of reactive oxygen species (ROS), mitochondrial dysfunction, and inflammation. Sustained AGE-RAGE signaling drives chronic inflammation contributing to the development of various ailments, including neurodegenerative diseases. This review examines AGE formation, metabolism, and accumulation, with an emphasis on dietary sources as modifiable contributors to AGE-RAGE mediated pathology. We highlight the need for further research on dietary AGE restriction as a potential strategy to prevent or slow the progression of neurodegenerative and neuroinflammatory disorders.

## 1. Introduction

It is estimated that more than 9 million people in the United States are currently living with a neurodegenerative disease (NDD), and, globally, neurological disorders affect approximately 15% of the population [1]. Myalgic encephalomyelitis/chronic fatigue syndrome (ME/CFS) is one such neurological condition that shares substantial clinical overlap with post-COVID syndrome (long COVID). In the United States, the ME/CFS patient population is estimated to be as high as 2.5 million [2,3]. ME/CFS is a complex and systemic disease, in which neuroinflammation is increasingly recognized as a central feature of its pathophysiology [4]. Chronic inflammation, elevated levels of reactive oxidative species (ROS), and sustained microglial activation are considered primary drivers for this multisystem illness. Although the molecular mechanisms underlying ME/CFS remain incompletely understood, recent findings [5] suggest that microglial activation observed in ME/CFS may be mediated, in part, by the interaction of advanced glycation end products (AGEs) with their receptor, RAGE. The interaction between AGEs and RAGE triggers pro-inflammatory intracellular signaling cascades. This activation of the AGE–RAGE axis leads to increased production of reactive oxygen species (ROS), mitochondrial dysfunction, and stimulation of the transcription factor NF-κB. Activated NF-κB, in turn, upregulates the expression of pro-inflammatory genes involved in microglial activation and neuroimmune response. Through these mechanisms, AGE–RAGE signaling contributes to chronic inflammation and aging and is considered a key driver in the development of various neurodegenerative diseases. Given the complexity of the AGE–RAGE pathway, it is often referred to as the AGE–RAGE axis, as it is feedback-driven, multifactorial, and not strictly linear.

AGEs in the human body can be produced endogenously or introduced externally through dietary sources. High blood sugar levels, as seen in diabetes, promote endogenous AGEs formation. Endogenous AGEs can form both intracellularly or extracellularly and have far-reaching effects, progressing from impaired cellular function to adverse impacts on tissue health. It is estimated that 10 to 30% of dietary AGEs are absorbed into the bloodstream following digestion, while the majority are unabsorbed and excreted via the gastrointestinal tract [6]. Although dietary AGEs represent a smaller fraction of the body’s total AGE burden, they contribute to elevated circulating AGE levels. Because cells express RAGE, circulating AGEs have the potential to form oligomers and activate the AGE–RAGE signaling. This activation promotes inflammation, oxidative stress, and mitochondrial dysfunction. The impact of AGE–RAGE signaling is profound, and AGEs are widely accepted as key contributors to aging and disease. Importantly, diet modification to reduce AGE intake has been shown to lower circulating AGE levels and decrease inflammation [7].

This review examines the biochemical formation of AGEs and their role in driving inflammation through the AGE–RAGE signaling axis, with a focus on the underlying mechanisms that contribute to neuroinflammatory processes. This review aims to elucidate the mechanisms by which AGEs, particularly those originating from Western dietary patterns and high-temperature cooking methods, contribute to chronic inflammation and the progression of neurodegenerative processes. By integrating molecular insights with emerging evidence on dietary modulation, we highlight the potential of AGE-restricted nutrition as a non-pharmacologic strategy to mitigate the neuroinflammatory load. While further clinical validation is needed, such interventions may offer benefits in reducing risk or attenuating symptoms associated with metabolic and neuroinflammatory conditions, including diabetes, Alzheimer’s disease, and post-viral syndromes such as long COVID.

## 2. Advanced Glycation End Products (AGEs): Formation and Accumulation

### 2.1. In Vitro Formation of AGEs-

**Figure 1:**
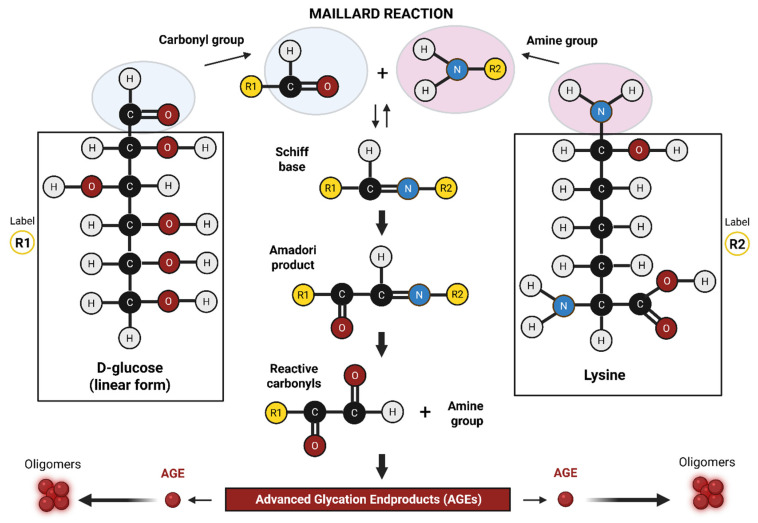
The Maillard reaction. This is a non-enzymatic reaction in which the carbonyl group of a reducing sugar (e.g., glucose) irreversibly binds the amine group of an amino acid (typically lysine or arginine) to form an AGE. Image was created using BioRender.

The Maillard reaction occurs in foods that are heated to high temperatures (above 140 °C or 300 °F), and is responsible for the browning commonly observed during the cooking process. First described in 1912 by French chemist Louis-Camille Maillard, the reaction was identified when he discovered that heating amino acids and sugars together caused a browning effect [8]. The mechanistic details of the Maillard reaction were later elucidated and described in 1953 by chemist John E. Hodge [9]. Examples of the Maillard reaction are encountered regularly in everyday life, ranging from grilled steaks (high protein, very low sugar) to toasted marshmallows (very low protein, high sugar). The Maillard reaction (Figure 1) is a sequence of non-enzymatic chemical reactions that occur when reducing sugars (e.g., glucose) react with amino acids, most commonly lysine and arginine residues in proteins, in the presence of heat. The reaction proceeds through well-characterized intermediates, involving the breaking and formation of new covalent bonds.

The initial step is the formation of a Schiff base between a carbonyl group of the reducing sugar and a free amino group of the protein. This step is reversible if conditions change (e.g., cooling or pH shift). If conditions persist, the Schiff base rearranges into a more stable intermediate known as an Amadori product. The formation of Amadori product involves the rearrangement of covalent bonds. This step is irreversible and ultimately leads to formation of AGEs. Importantly, Maillard reaction intermediates, such as the Amadori compound and the dicarbonyl species, also have the potential to react with lipids, further expanding the diversity and complexity of AGE structures.

### 2.2. In Vivo Formation of AGEs-

In 1968, Samuel Rahbar discovered glycated hemoglobin (HbA1c), providing evidence that the Maillard reaction occurs at a slow rate within the human body at body (37 °C) temperature [10]. In 1976, Anthony Cerami proposed the use of HbA1c as a diagnostic marker for diabetes [11,12] and introduced the term advanced glycosylation end products (AGEs).

A low level of AGE formation is a normal part of metabolism and occurs in all cell types and tissues. Endogenously formed AGEs are typically cleared through cellular quality control mechanisms such as the proteasome and lysosome. Low molecular weight AGE peptides generated extracellularly can enter the circulation and are usually cleared by the kidneys; however, some AGEs formed within the cell may accumulate over time, especially as the efficiency of the proteasome declines with age. When AGE accumulation reaches levels that impair cellular function, it can contribute to disease development.

Although AGE formation is a natural consequence of metabolism, it is markedly accelerated under pathological conditions such as diabetes, driven by hyperglycemia and oxidative stress [13]. Simply put, increased glucose availability raises the likelihood of the Maillard reaction occurring, as there is more substrate available for the reaction. This leads to elevated ROS production, increased AGE accumulation, cellular damage, mitochondrial dysfunction, and at last compromised tissue function (Table 1).

### 2.3. Tissue Storage and Accumulation of AGE

The end products of the Maillard reaction are irreversible, allowing AGEs to persist permanently in the body [14]. AGEs can form in nearly all cell types, including tissues with low protein abundance, such as adipose tissue and visceral fat [15]. At a cellular level, AGEs accumulate primarily in the extracellular matrix (ECM) through interactions with structural proteins such as collagens. Collagens are a common target of AGE crosslinking [16] and represent the most abundant protein family in the human body, accounting for approximately 30% of total protein mass [17]. Within cells, AGEs can bind to various proteins, but long-lived proteins [18,19] are especially prone to AGE accumulation and oligomerization over time. Under normal conditions, AGEs are cleared efficiently by cellular quality control systems; however, with aging or in disease states, the efficiency of these clearance mechanisms may decline. This decline can result in a build-up of intracellular AGE oligomers [20]. A primary concern arises when AGE deposits begin to impact cell function, which can ultimately compromise tissue function and overall health. It is now well established that AGEs gradually accumulate in the body over time, contributing to both aging and the development of chronic diseases.

## 3. Exogenous AGEs: Dietary Sources and Contributions

### 3.1. High-AGE Foods and Cooking Practices

While the body naturally produces AGEs, a significant portion is derived from dietary sources. The highest concentrations of AGEs are found in processed foods, meats, and cheeses, particularly when prepared using high-temperature dry-heat methods such as grilling, broiling, roasting, and frying [7]. In contrast, moist-heat cooking techniques, such as steaming, boiling, and poaching, generate significantly fewer AGEs [7]. It has been demonstrated that consuming a diet high in AGEs increases circulating AGE levels and inflammatory markers [7]. Although early beliefs were that dietary AGEs did not cross the gastrointestinal barrier and enter our bloodstream, meaning AGEs had no or little impact [21], compelling evidence now shows that reducing dietary AGEs can improve blood sugar regulation and reduce inflammation, particularly in patients with diabetes or renal disease [22,23]. Recent research has begun to explore the impact of dietary AGEs on brain health. Given their capacity to promote oxidative stress and chronic inflammation, AGEs have been linked with neurodegenerative diseases [24]. When AGEs accumulate in brain tissue, they can activate receptors like RAGE, triggering inflammatory cascades, fostering amyloid–beta (Aβ) aggregation, and impairing neuronal connectivity [18]. Over time, this may accelerate neuronal damage and contribute to dementia, including AD. Reducing AGE-rich foods may, therefore, represent a simple, accessible strategy to support brain health and delay cognitive decline [25].

### 3.2. Gastrointestinal Absorption and Organ Distribution

The standard American (Western) diet exposes individuals to ultra-processed foods containing dyes, preservatives, chemicals, microplastics, artificial flavorings, bleached and genetically modified ingredients, and agrochemicals, all of which contribute to a higher AGE load. High-temperature, dry-heat cooking further elevates AGEs levels in foods. Approximately 10% to 30% of dietary AGEs are now known to be absorbed through the gut [6]. Low-molecular-weight (LMW) AGEs enter the bloodstream by diffusion and are distributed to organs, including the liver, spleen, heart, kidneys, and lungs [25]. Larger, peptide-bound AGEs may also be actively transported across cell membranes.

Unabsorbed AGEs enter the colon, where gut microbiota attempt to metabolize them; those not metabolized are excreted in feces [6,25]. Excessive AGEs intake can further reduce both volume and diversity of beneficial bacteria (*Bacteroides*, *Bifidobacteria*, and *Lactobacilli*), disrupting gut health and contributing to metabolic disorders, chronic inflammation, immune dysfunction, insulin resistance, and non-alcoholic fatty liver diseases (NAFLD) [26,27]. Dysbiosis allows gram negative bacteria to release lipopolysaccharides (LPS) into circulation, activating TLR4 on microglia and triggering NF-κB-mediated inflammation, which disrupts BBB and fosters amyloid plaque deposition in AD [26,27,28]. This establishes a vicious cycle in which exogenous AGE consumption fuels further endogenous AGE production and systemic inflammation [23,25,27,28].

### 3.3. Renal Clearance and Implications for Neuroinflammation

Renal excretion is the primary pathway for clearing AGEs. Both exogenous and endogenously formed AGEs are filtered by the kidneys, with about one-third of circulating AGEs excreted within 48 h, while the remainder accumulates in the tissues [29,30,31,32]. AGE formation heightens in metabolic dysfunction states, such as diabetes and inflammation, and AGEs can accelerate kidney damage, creating a feedback loop that drives diabetic kidney disease [33]. The AGE clearance varies by type: LMW AGEs (e.g., carboxymethyl lysine, CML) are freely filtered and excreted, whereas high-molecular-weight protein-bound AGEs (e.g., glycated albumin) are poorly cleared and tend to accumulate, particularly in organs with slow protein turnover like the brain [31,32,33]. This accumulation poses serious risks for neuroinflammation and related damage. Moreover, medications are commonly used to treat neuroinflammatory diseases. such as non-steroidal anti-inflammatory drugs (NSAIDs), corticosteroids, immunosuppressants, and some cardiovascular drugs, can impair renal function, thereby worsening AGE clearance [34]. This underscores the need for careful consideration of renal health when managing neuroinflammatory conditions and designing therapeutic strategies to minimize AGE accumulation.

## 4. AGE–RAGE Signaling and Neuroinflammation

**Figure 2:**
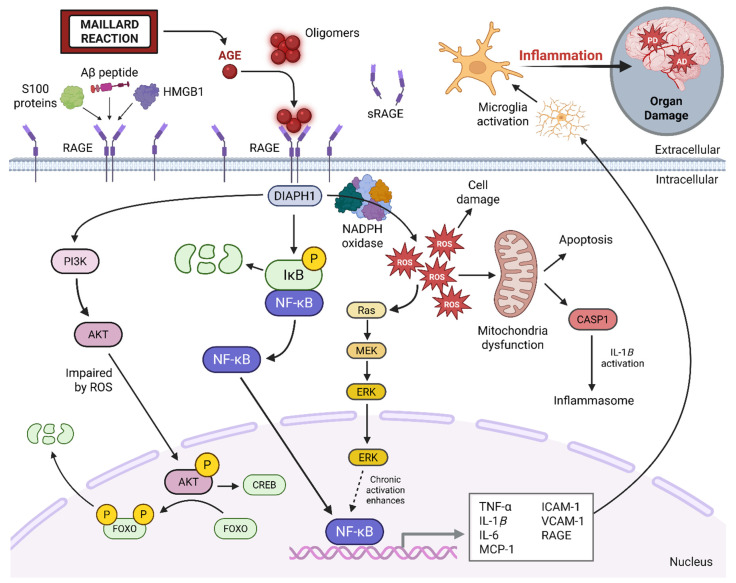
AGE–RAGE signaling axis. The AGE–RAGE signaling pathway is triggered when advanced glycation end products (AGEs) and other ligands (e.g., Aβ, S100, HMGB1) bind to the RAGE receptor. This interaction activates intracellular signaling through DIAPH1, leading to NF-κB activation and increased expression of pro-inflammatory genes. The pathway also increases reactive oxygen species (ROS) production, causing mitochondrial dysfunction and promoting further AGE formation. This creates a damaging feedback loop that impairs cell function and promotes chronic inflammation. Soluble RAGE (sRAGE) serves as a natural decoy, reducing pathway activation. Image was created using BioRender.

RAGE is a central mediator of AGE-induced inflammation and cellular dysfunction. In addition to binding AGEs, RAGE also serves as a receptor for other inflammation proteins such as high mobility group box 1 (HMGB1) and S100 family members [35]. Upon binding extracellular AGE oligomers, RAGE monomers may cluster on the cell membrane and undergo a conformational change. This structural modification allows the cytoplasmic domain of RAGE to engage in a direct protein–protein interaction with DIAPH1 (Diaphanous-related formin-1). DIAPH1 functions as a scaffold for the recruitment of adaptor proteins that initiates a cascade of proinflammatory intracellular signaling pathways (Figure 2).

A key downstream consequence of AGE–RAGE binding is the activation of nuclear factor kappa-light-chain-enhancer of activated B cells (NF-κB), a transcription factor that promotes the expression of multiple pro-inflammatory cytokines and mediators. These inflammatory signals, once secreted, can act in a paracrine fashion to activate resting microglia, the brain’s resident immune cells. Sustained microglial activation contributes to a persistent pro-inflammatory environment, which is increasingly recognized as a key driver in the pathogenesis of neurodegenerative disorders such as Alzheimer’s disease (AD). A growing body of evidence supports a mechanistic link between AGE–RAGE signaling in both Type 2 diabetes and AD (see Table 2). Due to overlapping features—including insulin resistance, oxidative stress, and chronic inflammation—the AGE–RAGE pathway has been implicated in the concept of ‘Type 3 diabetes,’ a term used to describe the metabolic and inflammatory parallels between Type 2 diabetes and AD [36].

Beyond its pro-inflammatory effects, the interaction between AGEs and RAGE leads to elevated production of reactive oxygen species (ROS), which directly damages cellular components such as proteins, lipids, and DNA. This oxidative stress also impairs mitochondrial function, initiating a feed-forward loop of ROS generation and mitochondrial dysfunction. The resulting oxidative environment further accelerates endogenous AGE formation, establishing a self-amplifying cycle of inflammation, oxidative stress, and AGE accumulation. Over time, intracellular AGE buildup may compromise cellular function and viability, particularly in terminally differentiated cells like neurons. Additionally, AGE modification of structural proteins—such as cytoskeletal elements and extracellular matrix components—disrupts cellular integrity and impairs intercellular communication.

Although endogenous defense mechanisms exist, such as the production of soluble RAGE (sRAGE), a decoy receptor that binds AGEs and prevents membrane-bound RAGE activation, these compensatory pathways are often insufficient or overwhelmed in chronic disease states. Consequently, the AGE–RAGE axis functions as a central mediator linking metabolic dysregulation to neuroinflammation and tissue injury. Elucidating and targeting this pathological loop through dietary modification and therapeutic intervention offers promising avenues for mitigating neurodegenerative and metabolic disease burden.

## 5. Post-Viral Syndromes and the AGE–RAGE Axis

### 5.1. AGE–RAGE Signaling in Post-Viral Disease: A Mechanistic Proposal

Post-viral syndromes, such as myalgic encephalomyelitis/chronic fatigue syndrome (ME/CFS) and long COVID, are increasingly recognized as chronic conditions that persist well beyond the resolution of the initial viral infection. These syndromes are characterized by symptoms including cognitive impairment (‘brain fog’), debilitating fatigue, chronic pain, and autonomic dysfunction. Mechanistically, they are thought to result from sustained immune activation, neuroinflammation, and mitochondrial dysfunction [37]. Recent evidence implicates the AGE–RAGE signaling axis as a potential contributor to the pathophysiology of post-viral syndromes. RAGE, a pattern recognition receptor expressed on various cell types, including endothelial cells, macrophages, and glial cells, is markedly upregulated during both acute and chronic viral infections. Upon binding to AGEs, the cytoplasmic domain of RAGE interacts with Diaphanous-1 (DIAPH1), initiating downstream signaling pathways such as MAPK (ERK1/2), PI3K/AKT, and JAK/STAT [38]. Activation of these cascades leads to the transcription of pro-inflammatory and pro-apoptotic genes [39], potentially sustaining the immune dysregulation and cellular stress observed in post-viral syndromes. Preclinical studies have shown that genetic deletion of RAGE or its pharmacological inhibition leads to improved outcomes in models of sepsis and influenza by dampening cytokine storms and preserving tissue integrity [40]. These findings support the role of RAGE as a molecular amplifier of post-viral inflammation. RAGE is abundantly expressed in alveolar epithelial cells, macrophages, and vascular endothelial cells, key sites of injury during respiratory viral infections. Upon activation by AGEs, RAGE initiates a hyperinflammatory response, contributing to complications such as acute respiratory distress syndrome (ARDS) [41,42]. In influenza models, RAGE deficiency enhances viral clearance and improves survival, underscoring the detrimental impact of excessive AGE–RAGE signaling in sustaining inflammatory responses [43].

Additionally, Epstein–Barr virus (EBV), commonly reactivated in ME/CFS and long COVID, expresses immunomodulatory proteins that alter host signaling pathways. For example, EBV-encoded dUTPase activates the NF-κB pathway via TLR2/MyD88, a signaling route that also lies downstream of RAGE [44,45]. These parallel signals converge to promote reactive oxygen species (ROS) production, pro-inflammatory cytokine release, and endothelial dysfunction [46], potentially compounding immune dysregulation in post-viral states. Emerging evidence suggests that Epstein–Barr virus (EBV) infection upregulates the expression of high mobility group box 1 (HMGB1), a well-characterized ligand of RAGE, thereby indirectly enhancing RAGE-mediated signaling [47,48]. Furthermore, EBV’s latent membrane protein 1 (LMP1) activates the PI3K/AKT and MAPK pathways in B cells and epithelial cells [47,48], signaling cascades that are similarly engaged downstream of RAGE activation [47,48,49]. The activation of these overlapping pro-inflammatory pathways may converge on NF-κB, resulting in synergistic amplification of its transcriptional activity and contributing to mitochondrial dysfunction. In nasopharyngeal carcinoma models, EBV-driven expression of RAGE ligands has been associated with poorer clinical outcomes and increased cellular invasiveness, potentially due to a feed-forward loop involving RAGE activation, reactive oxygen species (ROS), and hypoxia-inducible factor 1-alpha (HIF-1α) [50]. While direct biochemical interactions between EBV proteins and RAGE have not yet been demonstrated, the convergence of their intracellular signaling networks provides mechanistic plausibility for RAGE involvement in EBV-associated systemic inflammation. These observations highlight an emerging area of investigation and underscore the need for future functional studies to clarify these interactions in the context of post-viral syndromes.

### 5.2. SARS-CoV-2 and AGE–RAGE Signaling

Severe acute respiratory syndrome coronavirus 2 (SARS-CoV-2), the virus responsible for COVID-19, induces a sustained pro-inflammatory state that mechanistically overlaps with the AGE–RAGE signaling axis. During acute infection, SARS-CoV-2 triggers a strong cytokine response, endothelial dysfunction, and mitochondrial stress, all features characteristic of RAGE-mediated pathology [4,51,52,53,54]. RAGE is highly expressed in pulmonary epithelial cells, alveolar macrophages, and vascular endothelial cells, making these tissues particularly susceptible to SARS-CoV-2–induced injury [38,39]. Importantly, SARS-CoV-2 structural components, including the spike protein and viral RNA, activate toll-like receptors (TLR2 and TLR4), which converge on NF-κB signaling, paralleling the downstream pathways activated by AGE–RAGE interactions [53,55,56]. This convergence suggests that SARS-CoV-2 may exacerbate pre-existing RAGE signaling or sensitize tissues to AGE-related damage. Elevated circulating levels of AGEs and RAGE ligands, such as HMGB1 and S100 proteins, have been reported in severe COVID-19 cases and are associated with adverse outcomes, including acute respiratory distress syndrome (ARDS) and thromboinflammatory complications [55].

In the post-acute phase, persistent viral fragments and immune dysregulation in individuals with long COVID continue to drive oxidative stress through the generation of reactive oxygen species (ROS) [56], which enhance endogenous AGE formation and prolong RAGE activation. Moreover, SARS-CoV-2 has been shown to impair the integrity of the blood–brain barrier (BBB) [55,56,57], increasing central nervous system (CNS) permeability and potentially facilitating the entry of circulating AGEs into the brain. This may partially underlie the cognitive dysfunction and neuroinflammatory signatures observed in long COVID, which resemble those seen in neurodegenerative conditions such as Alzheimer’s disease and ME/CFS. Emerging evidence also implicates SARS-CoV-2 in impairing renal filtration efficiency, a key process for systemic AGE clearance, which may further exacerbate AGE accumulation [56]. Inflammatory injury to the glomeruli and tubular epithelium, commonly observed in post-acute COVID-19 sequelae, can prolong the circulating half-life of AGEs and increase their deposition in peripheral tissues, including the brain [57].

Collectively, these findings suggest that SARS-CoV-2 not only activates but may also amplify AGE–RAGE signaling, establishing a feed-forward loop of inflammation, oxidative stress, and tissue injury. This pathological cycle may contribute to the persistence of symptoms in long COVID and elevate the risk for neurodegenerative complications. These observations underscore the importance of exploring RAGE antagonists, AGE-reducing dietary strategies, and antioxidant therapies as potential interventions for mitigating post-viral complications and supporting recovery in individuals affected by COVID-19.

## 6. The Western Diet, AGE Formation, and Neurodegeneration

### 6.1. Western Diet as a Source of Exogenous and Endogenous AGEs

The Western diet, characterized by high consumption of red and processed meats, refined sugars, saturated fats, and ultra-processed foods, is a significant contributor to both exogenous and endogenous advanced glycation end-products (AGEs). Exogenous AGEs are introduced through dietary intake, particularly from foods prepared using high-temperature, dry-heat cooking methods such as grilling, frying, roasting, and broiling [7]. In contrast, endogenous AGEs are generated metabolically under conditions of chronic hyperglycemia, oxidative stress, and lipid peroxidation [15,19,20,21], all of which are frequently induced by Western dietary patterns. Processed meats and animal-derived foods are especially rich in pre-formed AGEs, as they contain abundant proteins and lipids that readily undergo Maillard reactions during high-heat cooking. These non-enzymatic reactions between reducing sugars and amino groups yield reactive carbonyl intermediates, which further react to form stable AGEs. Concurrently, the Western diet promotes a metabolic environment favorable to endogenous AGE production. Postprandial hyperglycemia, driven by refined carbohydrate intake, increases intracellular glucose flux through glycolytic and polyol pathways, leading to the generation of reactive carbonyl species (RCS) such as glyoxal, methylglyoxal, and 3-deoxyglucosone [58,59]. These RCS are potent glycation agents that modify intracellular and extracellular proteins, lipids, and nucleic acids.

Fructose, commonly found in sweetened beverages and high-fructose corn syrup, is particularly reactive and accelerates AGE formation more rapidly than glucose under physiological conditions [58]. This drives enhanced protein crosslinking, oxidative stress, and inflammatory signaling [59]. Moreover, the Western diet impairs endogenous antioxidant defenses. High intake of saturated fats disrupts mitochondrial function, reduces the activity of antioxidant enzymes such as superoxide dismutase (SOD) and increases reactive oxygen species (ROS) production [60]. This oxidative stress not only accelerates AGE generation but also activates redox-sensitive pathways, including RAGE-mediated inflammatory cascades.

In contrast, dietary patterns such as the Mediterranean and plant-based diets are associated with lower AGE burden. These diets emphasize whole, minimally processed foods, such as fruits, vegetables, legumes, and whole grains, and utilize cooking methods like steaming, boiling, or consuming foods raw, all of which limit exogenous AGE formation. Furthermore, these diets are rich in polyphenols and antioxidants [23], which can inhibit AGE formation and neutralize reactive intermediates, offering protective effects against glycation-induced oxidative stress and inflammation.

### 6.2. Environmental Contributors

In addition to high-heat cooking methods, the pro-glycation potential of the Western diet is exacerbated by environmental contaminants. Pesticide residues, plasticizers such as bisphenol A, heavy metals, and synthetic additives found in ultra-processed foods contribute to oxidative stress and mitochondrial dysfunction [61,62,63,64]. These environmental agents may synergize with dietary AGEs to potentiate RAGE activation and its downstream inflammatory signaling. Recent studies have shown that exposure to toxic heavy metals, particularly cadmium (Cd) and mercury (Hg), promotes AGE formation and amplifies RAGE-mediated inflammation [63,64]. In glycation system models, cadmium enhances the production of methylglyoxal (MGO) and hydrogen peroxide under hyperglycemic conditions, accelerating AGE generation from reactive carbonyl intermediates such as glyoxal and MGO [63,64]. Mechanistically, cadmium disrupts the glyoxalase detoxification system, leading to impaired clearance of these reactive carbonyl species and elevating endogenous AGE levels. Cd also induces mitochondrial dysfunction and oxidative stress, both of which intensify pro-inflammatory RAGE signaling. Moreover, cadmium damages renal glomeruli and tubular structures, reducing the kidney’s capacity to clear circulating AGEs and compounding systemic accumulation [64]. Similarly, mercury impairs redox balance and promotes mitochondrial injury, though its specific effects on glyoxalase activity and AGE formation are less well-characterized [65]. Nonetheless, both metals have been associated with heightened RAGE pathway activation, contributing to inflammation, apoptosis, and metabolic dysregulation. These metal-induced disruptions may synergize with dietary AGE burden to amplify systemic inflammation and neurotoxicity.

Beyond heavy metals, synthetic food additives, including dyes, preservatives, emulsifiers, and stabilizers, may further contribute to systemic oxidative stress and immune activation that intersect with AGE–RAGE signaling. Common artificial dyes such as tartrazine (Yellow No. 5) and Allura Red AC (Red No. 40) have been shown in animal models to increase oxidative stress and upregulate inflammatory gene expression [66]. These changes may indirectly promote endogenous AGE formation via mitochondrial dysfunction and carbonyl accumulation. Some synthetic dyes also compromise gut barrier integrity, potentially increasing systemic exposure to dietary AGEs and endotoxins. Preservatives like sodium benzoate, butylated hydroxytoluene (BHT), and nitrites can generate reactive oxygen species (ROS) and deplete glutathione stores, weakening cellular antioxidant defenses [67,68]. This pro-oxidative milieu facilitates non-enzymatic glycation reactions and elevates AGE burden, especially when these additives are consumed alongside high-sugar or high-fat foods. Additionally, stabilizers and emulsifiers such as carboxymethylcellulose and polysorbate-80 have been shown to alter gut microbiota composition and impair mucosal barrier function, contributing to low-grade systemic inflammation [69]. Such chronic inflammatory states may upregulate RAGE expression and sensitize tissues to AGE-mediated signaling.

Although direct mechanistic evidence linking these additives to AGE–RAGE activation is currently limited, their well-documented effects on redox balance, gut dysbiosis, and immune modulation support their potential role as cofactors in AGE-related pathologies. Given the widespread consumption of processed foods, further investigation into the cumulative and synergistic effects of these compounds with AGEs and RAGE ligands is warranted.

## 7. Pharmacological and Lifestyle Interventions

### 7.1. RAGE-Targeting Therapies: Current Limitations

Pharmacological strategies targeting AGE–RAGE signaling have shown promise in preclinical models, but clinical translation remains limited. Azeliragon (TTP488), an oral RAGE antagonist, progressed to Phase III trials for mild AD, after promising Phase II results, but failed to meet primary endpoints in the larger (STEADFAST) trial, though a diabetic subgroup showed cognitive benefits [70]. Azeliragon is now being explored for glioblastoma and metastatic breast cancer, due to RAGE’s role in tumor biology [71].

FPS-ZM1, another RAGE inhibitor, blocks NF-κB activation and has shown neuroprotective effects in rodent models of AD and chronic inflammation [72,73]. Alternative interventions, such as soluble RAGE (sRAGE) and anti-RAGE antibodies, which act as decoys to sequester ligands, have been effective in animal models but untested in humans [73]. Non-specific pharmacological agents such as metformin, statins, and thiazolidinediones have had limited success in reducing AGE burden [74]. Given the complexity of AGE–RAGE biology, combination therapies or lifestyle-based interventions, particularly dietary modification, may offer the most practical and effective path forward.

### 7.2. Dietary and Lifestyle Strategies to Reduce AGE Burden

Dietary and lifestyle choices play a critical role in modulating AGE accumulation and the associated inflammatory burden. Cooking methods are particularly influential; high-temperature techniques such as grilling and roasting significantly increase AGE content, whereas lower-temperature methods like boiling, steaming, or stewing minimize AGE formation [7]. Whole foods, especially vegetables, fruits, and legumes, are naturally low in AGEs. Additionally, marinating meats in acidic solutions (e.g., vinegar or lemon juice) has been shown to inhibit Maillard reaction chemistry, thereby reducing AGE generation during cooking [75,76].

Herbs and spices such as cinnamon, oregano, and cloves offer further protection through their antioxidant and antiglycation properties, largely attributed to their polyphenol content [77]. Environmental contaminants, including microplastics, mycotoxins from mold-contaminated foods, and pollutants, can also enhance oxidative stress and promote endogenous AGE production, reinforcing the importance of reducing both dietary and environmental exposures. High-AGE diets have been linked to accelerated cognitive decline, while AGE-restricted diets are associated with improvements in vascular function and reductions in neuroinflammation [78]. Mouse model studies further support that environmental toxins, including those found in processed foods and polluted environments, significantly elevate AGE formation and exacerbate RAGE-driven inflammatory signaling cascades [58,59,65,66].

When AGEs bind to their receptor (RAGE), they trigger intracellular signaling pathways that amplify oxidative stress, mitochondrial dysfunction, and chronic inflammation, hallmarks of neurodegeneration and metabolic decline. Therefore, lifestyle modifications that include careful ingredient selection, toxin avoidance, and optimized cooking methods can meaningfully reduce AGE–RAGE-mediated pathophysiology. Nutritional interventions that lower dietary AGE intake and attenuate RAGE activation show promise in preserving cognitive health and improving systemic inflammation. Anti-inflammatory diets, such as the Mediterranean and plant-based diets, which are rich in polyphenols and low in ultra-processed foods, have been shown to reduce circulating AGE levels and improve metabolic outcomes [79]. In contrast, the standard American diet (SAD), characterized by frequent consumption of processed foods and refined sugars, promotes postprandial glycemic spikes, and enhances endogenous AGE formation.

A comprehensive prevention strategy that emphasizes unprocessed foods, gentle cooking methods, antioxidant-rich ingredients, and environmental toxin reduction may mitigate AGE–RAGE-driven inflammation and reduce neurodegenerative risk. A 2025 population-based study using U.S. cohort data demonstrated strong correlations between elevated blood levels of cadmium, lead, and cigarette smoke metabolites with markers of accelerated biological aging [80]. As the AGE–RAGE axis is a central mediator of toxicant-induced stress and inflammaging, such exposures are believed to increase AGE production and RAGE expression, further amplifying oxidative and inflammatory cascades. These findings underscore the importance of integrative interventions that combine dietary, environmental, and lifestyle approaches to attenuate AGE–RAGE-related disease processes.

## 8. Conclusions and Future Directions

This review highlights the central role of AGEs and their interaction with RAGE in driving neuroinflammation and contributing to the pathogenesis of neurodegenerative and neuroinflammatory diseases, including AD, ME/CFS, and long COVID. These toxic AGE compounds, especially when introduced through the Western diet and high-heat cooking methods, initiate and sustain a damaging cycle of chronic inflammation, oxidative stress, and mitochondrial dysfunction that underpins progressive tissue injury and cognitive decline.

Unlike many fixed genetic or environmental risk factors, dietary AGEs represent a modifiable target, offering a powerful and practical opportunity for prevention and intervention. Simple changes in food choices, cooking methods, and nutritional awareness can substantially reduce AGE intake and may help interrupt the harmful AGE–RAGE signaling cascades. As our understanding of the biochemical consequences of diet deepens, the concept of “food as medicine” shifts from theory to necessity, positioning dietary intervention as a cornerstone of strategies to protect cognitive function and promote healthy aging.

Future research should prioritize rigorous dietary intervention trials in at-risk, aging, and post-viral populations to determine the impact of AGE-restricted diets on systemic inflammation, neuroinflammation, cognitive health, and metabolic balance. Combining these studies with molecular profiling of AGE–RAGE activity, advanced imaging, and biomarker development will enhance our understanding of individual susceptibility and inform precise nutrition strategies. Furthermore, integrating AGE education into public health policies, food labeling, nutritional guidelines, and community health initiatives is essential to empower individuals to make informed choices that support long-term brain health.

While pharmacologic approaches targeting the AGE–RAGE pathway have yet to achieve consistent success in clinical trials, the most immediate and accessible intervention remains within our daily choices: at the table, in the kitchen, and in our food systems. The greatest opportunity to safeguard cognitive vitality and slow the progression of neurodegenerative disease may lie not in the laboratory but on the plate.

## Figures and Tables

**Table 1:** Effects of AGEs on tissues during hyperglycemia as observed in diabetes.

Tissue	Why Vulnerable?	Example of AGE Effects
Kidneys	High glucose filtration; impaired clearance	Nephropathy, glomerular basement membrane thickening
Eyes	High metabolic rate; excessive glucose uptake	Retinopathy, blood–retinal barrier (BRB) breakdown
Peripheral nerves	High collagen content prone to AGE cross-linking	Atherosclerosis, vascular stiffening
Heart	High mitochondrial stress; susceptibility to inflammation	Diabetic cardiomyopathy
Brain	Low molecular weight AGE peptides cross the blood–brain barrier (BBB); circulating AGEs and RAGE at the BBB activating Nf-κB and microglia; increasing BBB permeability	Cognitive decline; increased Alzheimer’s disease risk

**Table 2:** AGE-RAGE in Type-2 Diabetes and Type-3 Alzheimer’s Disease.

Event	Diabetes (Type 2)	Alzheimer’s Disease (Type 3)
AGE accumulation	↑ due to chronic hyperglycemia	↑ in brain with aging; further elevated in diabetic individuals
RAGE activation	Chronic in vascular tissues	Chronic in neurons, glia, and cerebral vasculature
Inflammation/ROS	↑ vascular inflammation, oxidative stress	↑ neuroinflammation, mitochondrial ROS, redox imbalance
Protein aggregation	AGEs contribute to vascular and renal protein deposits	Aβ plaque formation & tau hyperphosphorylation
Clearance impairment	Impaired proteostasis and AGE clearance with diabetes/aging	Impaired clearance of Aβ and AGEs, exacerbated by diabetes-induced dysfunction

↑ depicts an increase/higher.

## References

[1] Feigin V.L., Nichols E., Alam T., Bannick M.S., Beghi E., Blake N., Culpepper W.J., Dorsey E.R., Elbaz A., Ellenbogen R.G. (2019). Global burden of neurological disorders, 1990–2016: A systematic analysis for the Global Burden of Disease Study 2016. Lancet Neurol..

[2] Sirotiak Z., Thomas E.B. (2025). Beyond fatigue: An intersectional analysis of myalgic encephalomyelitis/chronic fatigue syndrome (ME/CFS) and social identities. Curr. Psychol..

[3] Slavin M.D., Bailey H.M., Hickey E.J., Vasudevan A., Ledingham A., Tannenbaum L., Bateman L., Kaufman D.L., Peterson D.L., Ruhoy I.S. (2023). Myalgic Encephalomyelitis-Chronic Fatigue Syndrome Common Data Element item content analysis. PLoS ONE.

[4] VanElzakker M.B., Brumfield S.A., Lara Mejia P.S. (2019). Neuroinflammation and cytokines in myalgic encephalomyelitis/chronic fatigue syndrome (ME/CFS): A critical review of research methods. Front. Neurol..

[5] Gottschalk G., Peterson D., Knox K., Maynard M., Whelan R.J., Roy A. (2022). Elevated ATG13 in serum of patients with ME/CFS stimulates oxidative stress response in microglial cells via activation of receptor for advanced glycation end products (RAGE). Mol. Cell. Neurosci..

[6] Luevano-Contreras C., Chapman-Novakofski K. (2010). Dietary advanced glycation end products and aging. Nutrients.

[7] Uribarri J., Woodruff S., Goodman S., Cai W., Chen X.U., Pyzik R., Yong A., Striker G.E., Vlassara H. (2010). Advanced glycation end products in foods and a practical guide to their reduction in the diet. J. Am. Diet. Assoc..

[8] Maillard L.C. (1912). Action des acides amines sur les sucres: Formation des melanoidines par voie methodique. CR Seances Soc. Biol. Fil..

[9] Hodge J.E. (1953). Dehydrated foods, chemistry of browning reactions in model systems. J. Agric. Food Chem..

[10] Rahbar S. (1968). An abnormal hemoglobin in red cells of diabetics. Clin. Chim. Acta.

[11] Koenig R.J., Peterson C.M., Jones R.L., Saudek C., Lehrman M., Cerami A. (1976). Correlation of glucose regulation and hemoglobin AIc in diabetes mellitus. N. Engl. J. Med..

[12] Cerami A. (2012). The unexpected pathway to the creation of the HbA1c test and the discovery of AGE’s. J. Intern. Med..

[13] Ramasamy R., Vannucci S.J., Yan S.S., Herold K., Yan S.F., Schmidt A.M. (2005). Advanced glycation end products and RAGE: A common thread in aging, diabetes, neurodegeneration, and inflammation. Glycobiology.

[14] Sharma C., Kaur A., Thind S.S., Singh B., Raina S. (2015). Advanced glycation End-products (AGEs): An emerging concern for processed food industries. J. Food Sci. Technol..

[15] Gaens K.H., Goossens G.H., Niessen P.M., van Greevenbroek M.M., van der Kallen C.J., Niessen H.W., Rensen S.S., Buurman W.A., Greve J.W., Blaak E.E. (2014). Nε-(carboxymethyl) lysine-receptor for advanced glycation end product axis is a key modulator of obesity-induced dysregulation of adipokine expression and insulin resistance. Arterioscler. Thromb. Vasc. Biol..

[16] Verzijl N., DeGroot J., Oldehinkel E., Bank R.A., Thorpe S.R., Baynes J.W., Bayliss M.T., Bijlsma J.W., Lafeber F.P., TeKoppele J.M. (2000). Age-related accumulation of Maillard reaction products in human articular cartilage collagen. Biochem. J..

[17] Uceda A.B., Mariño L., Casasnovas R., Adrover M. (2024). An overview on glycation: Molecular mechanisms, impact on proteins, pathogenesis, and inhibition. Biophys. Rev..

[18] Twarda-Clapa A., Olczak A., Białkowska A.M., Koziołkiewicz M. (2022). Advanced glycation end-products (AGEs): Formation, chemistry, classification, receptors, and diseases related to AGEs. Cells.

[19] Simm A., Nass N., Bartling B., Hofmann B., Silber R.E., Navarrete Santos A. (2008). Potential biomarkers of ageing. Biol. Chem..

[20] Koschinsky T., He C.J., Mitsuhashi T., Bucala R., Liu C., Buenting C., Heitmann K., Vlassara H. (1997). Orally absorbed reactive glycation products (glycotoxins): An environmental risk factor in diabetic nephropathy. Proc. Natl. Acad. Sci. USA.

[21] Vlassara H., Uribarri J. (2014). Advanced glycation end products (AGE) and diabetes: Cause, effect, or both?. Curr. Diabetes Rep..

[22] Srikanth V., Maczurek A., Phan T., Steele M., Westcott B., Juskiw D., Münch G. (2011). Advanced glycation endproducts and their receptor RAGE in Alzheimer’s disease. Neurobiol. Aging.

[23] De la Monte S.M. (2017). Insulin resistance and neurodegeneration: Progress towards the development of new therapeutics for Alzheimer’s disease. Drugs.

[24] Zhang R., Jiang L., Li G., Wu J., Tian P., Zhang D., Qin Y., Shi Z., Gao Z., Zhang N. (2022). Advanced glycosylation end products induced synaptic deficits and cognitive decline through ROS-JNK-p53/miR-34c/SYT1 axis in diabetic encephalopathy. J. Alzheimer’s Dis..

[25] Snelson M., Coughlan M.T. (2019). Dietary advanced glycation end products: Digestion, metabolism and modulation of gut microbial ecology. Nutrients.

[26] Kook S.Y., Seok Hong H., Moon M., Mook-Jung I. (2013). Disruption of blood-brain barrier in Alzheimer’s disease pathogenesis. Tissue Barriers.

[27] Franceschi C., Garagnani P., Parini P., Giuliani C., Santoro A. (2018). Inflammaging: A new immune–metabolic viewpoint for age-related diseases. Nat. Rev. Endocrinol..

[28] Phuong-Nguyen K., McNeill B.A., Aston-Mourney K., Rivera L.R. (2023). Advanced glycation end-products and their effects on gut health. Nutrients.

[29] Palaseweenun P., Hagen-Plantinga E.A., Schonewille J.T., Koop G., Butre C., Jonathan M., Wierenga P.A., Hendriks W.H. (2021). Urinary excretion of advanced glycation end products in dogs and cats. J. Anim. Physiol. Anim. Nutr..

[30] Liang Z., Chen X., Li L., Li B., Yang Z. (2020). The fate of dietary advanced glycation end products in the body: From oral intake to excretion. Crit. Rev. Food Sci. Nutr..

[31] Koska J., Gerstein H.C., Beisswenger P.J., Reaven P.D. (2022). Advanced glycation end products predict loss of renal function and high-risk chronic kidney disease in type 2 diabetes. Diabetes Care.

[32] Roncero-Ramos I., Delgado-Andrade C., Tessier F.J., Niquet-Léridon C., Strauch C., Monnier V.M., Navarro M.P. (2013). Metabolic transit of Nε-carboxymethyl-lysine after consumption of AGEs from bread crust. Food Funct..

[33] Lüth H.J., Ogunlade V., Kuhla B., Kientsch-Engel R., Stahl P., Webster J., Arendt T., Münch G. (2005). Age-and stage-dependent accumulation of advanced glycation end products in intracellular deposits in normal and Alzheimer’s disease brains. Cereb. Cortex.

[34] Gooch K., Culleton B.F., Manns B.J., Zhang J., Alfonso H., Tonelli M., Frank C., Klarenbach S., Hemmelgarn B.R. (2007). NSAID use and progression of chronic kidney disease. Am. J. Med..

[35] Sparvero L.J., Asafu-Adjei D., Kang R., Tang D., Amin N., Im J., Rutledge R., Lin B., Amoscato A.A., Zeh H.J. (2009). RAGE (Receptor for Advanced Glycation Endproducts), RAGE ligands, and their role in cancer and inflammation. J. Transl. Med..

[36] Yang Y., Song W. (2013). Molecular links between Alzheimer’s disease and diabetes mellitus. Neuroscience.

[37] Komaroff A.L., Bateman L. (2021). Will COVID-19 lead to myalgic encephalomyelitis/chronic fatigue syndrome?. Front. Med..

[38] Manigrasso M.B., Pan J., Rai V., Zhang J., Reverdatto S., Quadri N., DeVita R.J., Ramasamy R., Shekhtman A., Schmidt A.M. (2016). Small molecule inhibition of ligand-stimulated RAGE-DIAPH1 signal transduction. Sci. Rep..

[39] Piperi C., Goumenos A., Adamopoulos C., Papavassiliou A.G. (2015). AGE/RAGE signalling regulation by miRNAs: Associations with diabetic complications and therapeutic potential. Int. J. Biochem. Cell Biol..

[40] Xiong X., Dou J., Shi J., Ren Y., Wang C., Zhang Y., Cui Y. (2023). RAGE inhibition alleviates lipopolysaccharides-induced lung injury via directly suppressing autophagic apoptosis of type II alveolar epithelial cells. Respir. Res..

[41] Oczypok E.A., Perkins T.N., Oury T.D. (2017). All the “RAGE” in lung disease: The receptor for advanced glycation endproducts (RAGE) is a major mediator of pulmonary inflammatory responses. Paediatr. Respir. Rev..

[42] Rojas A., Gonzalez I., Morales M.A. (2020). SARS-CoV-2-mediated inflammatory response in lungs: Should we look at RAGE?. Inflamm. Res..

[43] Bopp C., Bierhaus A., Hofer S., Bouchon A., Nawroth P.P., Martin E., Weigand M.A. (2008). Bench-to-bedside review: The inflammation-perpetuating pattern-recognition receptor RAGE as a therapeutic target in sepsis. Crit. Care.

[44] Ariza M.E., Glaser R., Kaumaya P.T., Jones C., Williams M.V. (2009). The EBV-encoded dUTPase activates NF-κB through the TLR2 and MyD88-dependent signaling pathway. J. Immunol..

[45] Ariza M.E., Rivailler P., Glaser R., Chen M., Williams M.V. (2013). Epstein-Barr virus encoded dUTPase containing exosomes modulate innate and adaptive immune responses in human dendritic cells and peripheral blood mononuclear cells. PLoS ONE.

[46] Ostrand-Rosenberg S., Huecksteadt T., Sanders K. (2023). The receptor for advanced glycation endproducts (RAGE) and its ligands S100A8/A9 and high mobility group box protein 1 (HMGB1) are key regulators of myeloid-derived suppressor cells. Cancers.

[47] Selvam R., Khan E., Mehta M.K., Singh D., Gupta S., Chandra S. (2024). Epstein-Barr virus-encoded Latent Membrane protein-1 (LMP-1) as a Prognostic marker in OSCC and OPMDs. Oral Oncol. Rep..

[48] Šimičić P., Batović M., Stojanović Marković A., Židovec-Lepej S. (2024). Deciphering the role of Epstein–Barr virus latent membrane protein 1 in immune modulation: A multifaced Signalling perspective. Viruses.

[49] Li S., Wang Y., Sun X., Lu L., Yong Y., Kong X., Song J. (2025). Suppressing neuroinflammation by Shenfu injection against ischemic stroke in mice via inhibiting RAGE-PI3K-Akt pathway. Phytomedicine.

[50] Liu Y., Lui K.S., Ye Z., Chen L., Cheung A.K. (2024). Epstein–Barr Virus BRRF1 Induces Butyrophilin 2A1 in Nasopharyngeal Carcinoma NPC43 Cells via the IL-22/JAK3-STAT3 Pathway. Int. J. Mol. Sci..

[51] Sivagurunathan N., Calivarathan L. (2024). SARS-CoV-2 infection to premature neuronal aging and neurodegenerative diseases: Is there any connection with Hypoxia?. CNS Neurol. Disord. Drug Targets.

[52] Shirato K., Kizaki T. (2021). SARS-CoV-2 spike protein S1 subunit induces pro-inflammatory responses via toll-like receptor 4 signaling in murine and human macrophages. Heliyon.

[53] van Beijnum J.R., Buurman W.A., Griffioen A.W. (2008). Convergence and amplification of toll-like receptor (TLR) and receptor for advanced glycation end products (RAGE) signaling pathways via high mobility group B1 (HMGB1). Angiogenesis.

[54] Shankar V., Wilhelmy J., Curtis E.J., Michael B., Cervantes L., Mallajosyula V., Davis R.W., Snyder M., Younis S., Robinson W.H. (2025). Oxidative stress is a shared characteristic of ME/CFS and Long COVID. Proc. Natl. Acad. Sci. USA.

[55] DeOre B.J., Tran K.A., Andrews A.M., Ramirez S.H., Galie P.A. (2021). SARS-CoV-2 spike protein disrupts blood–brain barrier integrity via RhoA activation. J. Neuroimmune Pharmacol..

[56] Martinez-Rojas M.A., Vega-Vega O., Bobadilla N.A. (2020). Is the kidney a target of SARS-CoV-2?. Am. J. Physiol.-Ren. Physiol..

[57] He W., Liu X., Hu B., Li D., Chen L., Li Y., Tu Y., Xiong S., Wang G., Deng J. (2022). Mechanisms of SARS-CoV-2 infection-induced kidney injury: A literature review. Front. Cell. Infect. Microbiol..

[58] Iqbal A., Hafeez Kamran S., Siddique F., Ishtiaq S., Hameed M., Manzoor M. (2024). Modulatory effects of rutin and vitamin A on hyperglycemia induced glycation, oxidative stress and inflammation in high-fat-fructose diet animal model. PLoS ONE.

[59] Levi B., Werman M.J. (1998). Long-term fructose consumption accelerates glycation and several age-related variables in male rats. J. Nutr..

[60] de Lima N.C., Fernandes-Batista T., Ferreira-Serra L., Paes-Dias A.L., Matta-Pereira L., Medeiros F.H., Pazos-Moura C.C., Fortunato R.S., Carvalho D.P., Dias G.R. (2025). Oxidative Stress Parameters are Differentially Regulated in Visceral and Subcutaneous Adipose Tissue by Western Diet and Intermittent Fasting. Horm. Metab. Res..

[61] Huang W., Zhang Z., Colucci M., Deng L., Yang M., Huang X., Zhou X., Jin Y., Lazzarini E., Balbi C. (2024). The mixed effect of Endocrine-Disrupting chemicals on biological age Acceleration: Unveiling the mechanism and potential intervention target. Environ. Int..

[62] Skalny A.V., Aschner M., Santamaria A., Lu R., Rocha J.B., Zalavina S.V., Korchin V.I., Ke T., Tinkov A.A. (2024). The Differential Role of Toxic and Essential Metals in Formation and Toxicity of Advanced Glycation End-Products. Toxicology of Essential and Xenobiotic Metals.

[63] Suhartono E., Triawanti A.S., Djati M.S. (2014). The Role of Cadmium in Proteins Glycation by Glucose: Formation of Methylglyoxal and Hydrogen Peroxide In Vitro. J. Med. Bioeng..

[64] Wu H., Liao Q., Chillrud S.N., Yang Q., Huang L., Bi J., Yan B. (2016). Environmental exposure to cadmium: Health risk assessment and its associations with hypertension and impaired kidney function. Sci. Rep..

[65] Ma L., Bi K.D., Fan Y.M., Jiang Z.Y., Zhang X.Y., Zhang J.W., Zhao J., Jiang F.L., Dong J.X. (2018). In vitro modulation of mercury-induced rat liver mitochondria dysfunction. Toxicol. Res..

[66] Wu L., Xu Y., Lv X., Chang X., Ma X., Tian X., Shi X., Li X., Kong X. (2021). Impacts of an azo food dye tartrazine uptake on intestinal barrier, oxidative stress, inflammatory response and intestinal microbiome in crucian carp (*Carassius auratus*). Ecotoxicol. Environ. Saf..

[67] Gupta R., Yadav R.K. (2021). Impact of chemical food preservatives on human health. Palarch’s J. Archaeol. Egypt/Egyptol..

[68] Mandal D. (2019). Food preservative chemistry: Effects and side effects. J. Indian Chem. Soc..

[69] Naimi S., Viennois E., Gewirtz A.T., Chassaing B. (2021). Direct impact of commonly used dietary emulsifiers on human gut microbiota. Microbiome.

[70] Burstein A.H., Sabbagh M., Andrews R., Valcarce C., Dunn I., Altstiel L. (2018). Development of Azeliragon, an oral small molecule antagonist of the receptor for advanced glycation endproducts, for the potential slowing of loss of cognition in mild Alzheimer’s disease. J. Prev. Alzheimer’s Dis..

[71] Magna M., Hwang G.H., McIntosh A., Drews-Elger K., Takabatake M., Ikeda A., Mera B.J., Kwak T., Miller P., Lippman M.E. (2023). RAGE inhibitor TTP488 (Azeliragon) suppresses metastasis in triple-negative breast cancer. NPJ Breast Cancer.

[72] Liu Y., Shen W., Chen Q., Cao Q., Di W., Lan R., Chen Z., Bai J., Han Z., Xu W. (2020). Inhibition of RAGE by FPS-ZM1 alleviates renal injury in spontaneously hypertensive rats. Eur. J. Pharmacol..

[73] Shen L., Zhang T., Yang Y., Lu D., Xu A., Li K. (2020). FPS-ZM1 alleviates neuroinflammation in focal cerebral ischemia rats via blocking ligand/RAGE/DIAPH1 pathway. ACS Chem. Neurosci..

[74] Dong H., Zhang Y., Huang Y., Deng H. (2022). Pathophysiology of RAGE in inflammatory diseases. Front. Immunol..

[75] Inan-Eroglu E., Ayaz A., Buyuktuncer Z. (2020). Formation of advanced glycation endproducts in foods during cooking process and underlying mechanisms: A comprehensive review of experimental studies. Nutr. Res. Rev..

[76] Goldberg T., Cai W., Peppa M., Dardaine V., Baliga B.S., Uribarri J., Vlassara H. (2004). Advanced glycoxidation end products in commonly consumed foods. J. Am. Diet. Assoc..

[77] Wu C.H., Yen G.C. (2005). Inhibitory effect of naturally occurring flavonoids on the formation of advanced glycation endproducts. J. Agric. Food Chem..

[78] Poznyak A.V., Sukhorukov V.N., Surkova R., Orekhov N.A., Orekhov A.N. (2023). Glycation of LDL: AGEs, impact on lipoprotein function, and involvement in atherosclerosis. Front. Cardiovasc. Med..

[79] Tsigalou C., Konstantinidis T., Paraschaki A., Stavropoulou E., Voidarou C., Bezirtzoglou E. (2020). Mediterranean diet as a tool to combat inflammation and chronic diseases. An overview. Biomedicines.

[80] Fang Y.W., Chen C.W., Su T.C., Wang C., Lin C.Y. (2025). Investigating the associations of blood lead and cadmium with smoking-related DNA methylation and mortality among US adults. Ecotoxicol. Environ. Saf..

